# Brownian Sieving Effect for Boosting the Performance of Microcapillary Hydrodynamic Chromatography. Proof of Concept

**DOI:** 10.1021/acs.analchem.1c00780

**Published:** 2021-04-23

**Authors:** Valentina Biagioni, Alpha L. Sow, Alessandra Adrover, Stefano Cerbelli

**Affiliations:** Dipartimento di Ingegneria Chimica Materiali Ambiente, Sapienza Università di Roma, Via Eudossiana 18, Roma 00184, Italy

## Abstract

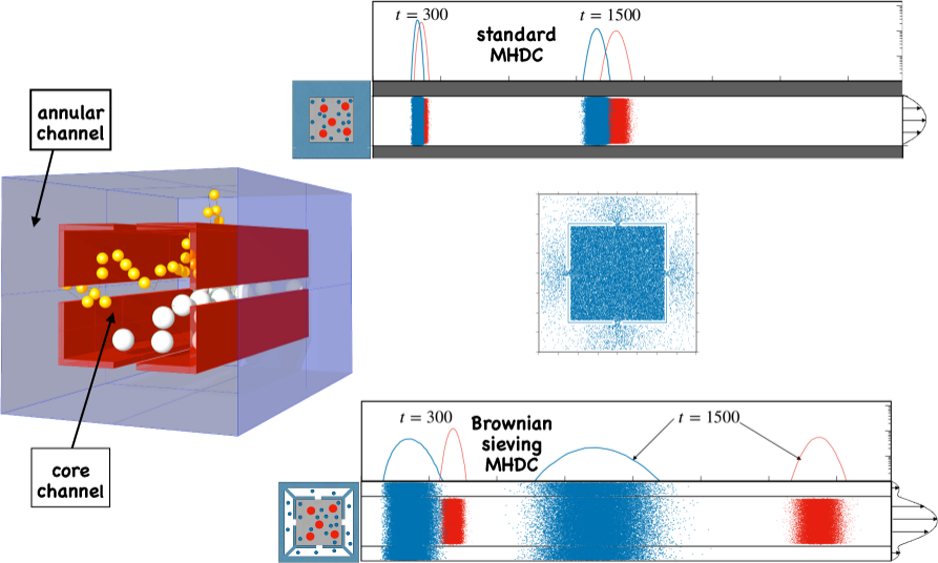

Microcapillary hydrodynamic chromatography (MHDC) is a well-established technique for the size-based separation of suspensions and colloids, where the characteristic size of the dispersed phase ranges from tens of nanometers to micrometers. It is based on hindrance effects which prevent relatively large particles from experiencing the low velocity region near the walls of a pressure-driven laminar flow through an empty microchannel. An improved device design is here proposed, where the relative extent of the low velocity region is made tunable by exploiting a two-channel annular geometry. The geometry is designed so that the core and the annular channel are characterized by different average flow velocities when subject to one and the same pressure drop. The channels communicate through openings of assigned cut-off length, say *A*. As they move downstream the channel, particles of size bigger than *A* are confined to the core region, whereas smaller particles can diffuse through the openings and spread throughout the entire cross section, therein attaining a spatially uniform distribution. By using a classical excluded-volume approach for modeling particle transport, we perform Lagrangian-stochastic simulations of particle dynamics and compare the separation performance of the two-channel and the standard (single-channel) MHDC. Results suggest that a quantitative (up to thirtyfold) performance enhancement can be obtained at operating conditions and values of the transport parameters commonly encountered in practical implementations of MHDC. The separation principle can readily be extended to a multistage geometry when the efficient fractionation of an arbitrary size distribution of the suspension is sought.

## Introduction

The size-based separation of particle suspensions or colloidal matter is a subject of wide interest in a variety of analytical methods, ranging from clinical/biological essays^1,2^ to polymer characterization,^3^ encompassing food^4^ and environmental engineering^5−7^ applications. Often, multiple detectors are used in line downstream the separation unit^8^ so that the size of the suspended particles can be cross-correlated with other fundamental properties of the dispersed phase such as particle number density, shape, mass and thermal diffusion coefficient, hydrodynamic diameter, equivalent spherical volume diameter, zeta potential, electrophoretic mobility, molecular structure, and molecular weight distribution (see, e.g., refs (9) and (10) and therein cited literature).

A number of flow-based different techniques have been proposed as separation methods for noncharged suspensions, which can be grouped into two broad categories, namely, liquid chromatography^11^ (LC) and field-flow-fractionation^12^ (FFF). In both cases, the suspension or colloid is flown with an eluent through a column or capillary. In LC, the column may or may not contain a stationary phase, whereas an empty channel geometry is typically used in FFF. In addition to the main streamwise component of the flow, FFF methods combine an active external force directed along the orthogonal direction to the channel axis, which is responsible for particle separation.^13^ Owing to the variety of choices for the physical field driving the separation, FFF is widely recognized as a versatile and highly selective technique. Besides, the fine tuning of operating conditions and of the channel geometry of FFF-based separations may sensitively be dependent on the specific analytical target, a feature that makes their practical implementation not always straightforward.

LC-based methods, on the other hand, hinge on purely passive transport of the dispersed phase by convection and diffusion and on its interaction with the solid surfaces confining the flow. As such, they are conceptually simpler than FFF and may prove to be convenient in terms of operating ease. LC techniques can be subdivided into two main classes, namely, size exclusion chromatography^14^ (SEC) and hydrodynamic chromatography^15^ (HDC), which are primarily based on different separation mechanisms.

In SEC, the column is packed with porous grains of given characteristic pore diameter, say *d*_g_. Suspended particles whose size falls below *d*_g_ can enter the grains by diffusion, whereas particles of size bigger than *d*_g_ are confined to the mobile phase and are eluted first. Among the intrinsic issues that can hinder the operational simplicity and readiness of SEC analysis, one can recognize giant axial dispersion of particles smaller than the cut-off length *d*_g_, high operating pressure, and large shear forces associated with the high operating pressure, which can impact upon the shape/integrity of the analyte. Also, adsorption phenomena may occur alongside analyte transport, which may overshadow a strictly size-based sorting criterion.

**Figure 1:**
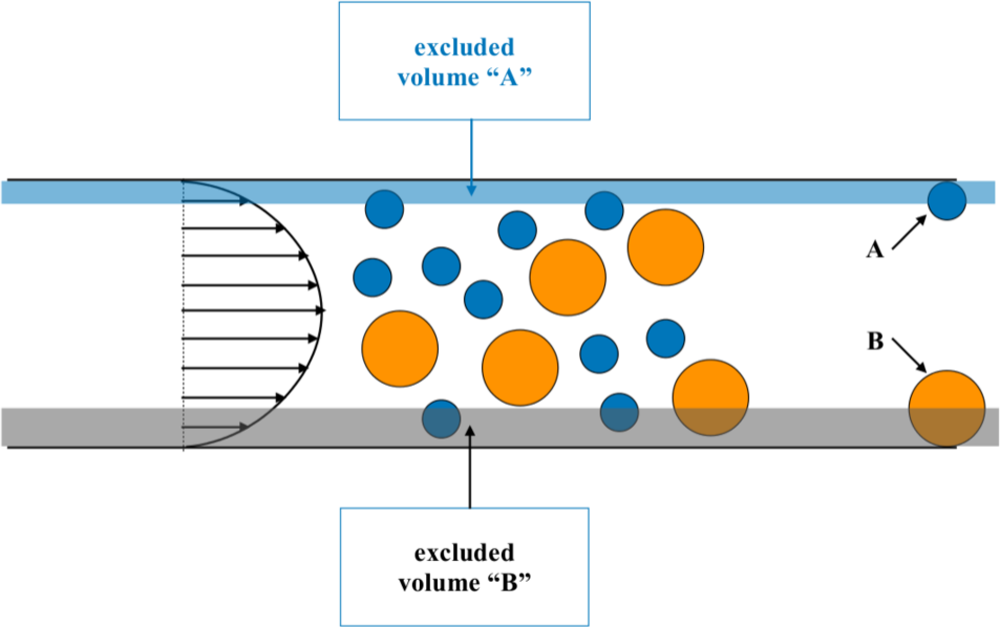
Schematic representation of the size-based separation mechanism in microcapillary HDC. The gray- and blue-shaded regions near the wall of the cylindrical capillary represent the excluded volume unaccessible to the center of the large and small particles, respectively.

Unlike SEC, HDC is not based on the distribution of the analyte between the mobile and the stationary phase but rather on the existence of a nonuniform axial velocity profile, which stretches out over the entire the channel cross section due to the prevailing laminar regime characterizing microchannel flow. The driving force for the separation is here based on the fact that the size, say *d*_p_, of the suspended particles is not altogether negligible with respect to the characteristic linear dimension of the channel cross section. Specifically, consider the case of an empty cylindrical capillary where a Poiseuille pressure-driven velocity profile of the eluent has been established (see Figure 1).

If two-phase effects and particle inertia can be neglected (the so-called one-way-coupling approximation), it can be assumed that the instantaneous velocity of the particle matches the velocity of the single-phase laminar flow computed at the particle center of mass. Besides, if the particle residence time is much bigger than the diffusive timescale over the channel cross section, because of the presence of diffusional motion, the particle will visit, with equal frequency, all the positions of the cross section that are allowed to be occupied by its center of mass, thereby performing an ergodic (i.e. uniform) average of the velocity profile restricted to the accessible region over the cross section. Since the extent of the low velocity region inhibited to the particle center depends on the particle size, so does the average particle velocity. As a result, the average particle velocity in the channel is an increasing function of the particle size. For an open cylindrical capillary, this dependence can be made explicit analytically in the form
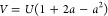
1where *V* is the average particle velocity, *U* is the average velocity of the eluent, and *a* = *d*_p_/(2*R*_c_) is the ratio of the particle diameter, *d*_p_, to the capillary diameter 2*R*_c_, *R*_c_ being the radius of the capillary. A qualitatively similar dependence is observed in rectangular channels, which may prove more suitable for being integrated in μ-TAS devices.^16,17^ Laminar flows in open channels have also been used to resolve mixtures of small molecules, either exploiting significant differences in the bare diffusion coefficient of the species or else by entraining molecular aggregates in the flow which possess different affinity toward the molecular species.^18,19^ Clearly, similar to the case of SEC-based separations, convection-amplified dispersion effects are also present in HDC, yet they are generally less severe and can be reduced by wall patterning strategies.^20,21^ More importantly, when open channel geometries are being used, the axial dispersion coefficients can be estimated through affordable analytical/numerical approaches,^22,23^ thus allowing to predict the separation performance with accuracy. In what follows, we refer to HCD separations exploiting open channel geometries as MHDC (microcapillary hydrodynamic chromatography). One major drawback of MHDC is represented by its low selectivity, meaning that the dependence of the average particle velocity on particle size is typically weak, as can be gathered, for example, by the relationship expressed by eq 1 for the case of cylindrical capillaries. This dependence can be ultimately pinned to how rapidly the fluid velocity increases when moving away from the channel walls toward the core of the channel. In standard (MHDC) separations, this feature is fixed by the shape of the cross section, typically either rectangular or circular, which yields a maximum difference between particles of different size of order 10 ÷ 15% in typical conditions. This low selectivity forces to use exceedingly long channels, increasing the operational time of the analysis and the pressure drop, thus confining the operational range of MHDC to low throughput processes. To overcome this shortcoming, combinations between MHDC and SEC and MHDC and FFF methods as well as MHDC coupled with electrokinetic flows have been proposed.^24−27^

**Figure 2:**
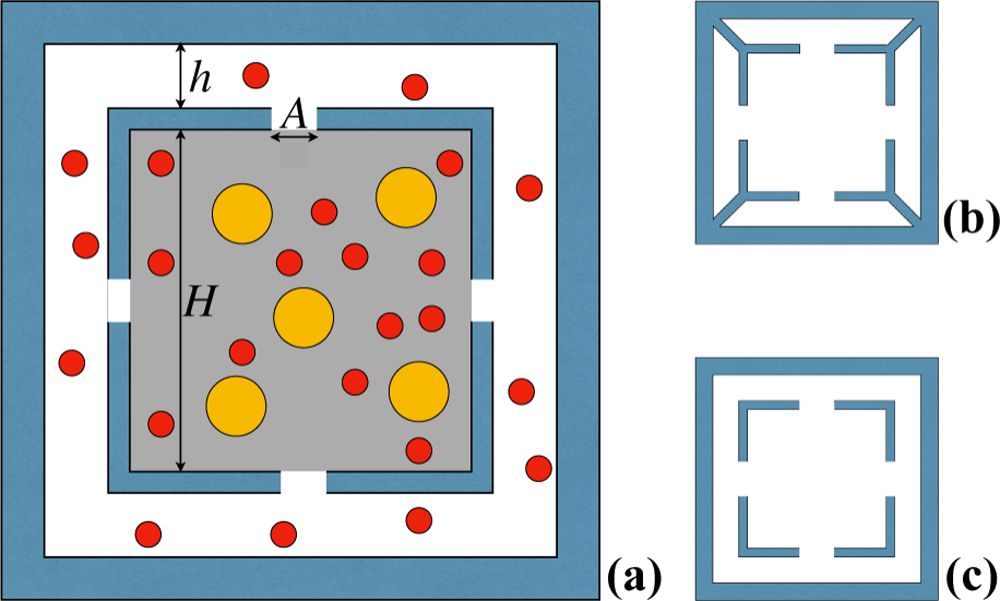
Schematic representation of the two-channel cross-section geometry enforcing the Brownian sieving mechanism for particles smaller than the cut-off length *A*; panel (a): relevant geometric parameters and relative particle size. The internal channel is held in place by alternately extruding the cross-sectional profiles depicted in panels (b,c). By appropriately tuning the ratio *h*/*H*, the velocity difference between particles below and above the cut-off opening length *A* can be amplified with respect to the standard MHDC.

From the above observations, it is readily understood that a great benefit could be gained in the enhancement of MHDC performance if the shape of the near-wall velocity profile could be tuned up unconstrainedly, so that maximum selectivity could be tailored to a specific particle size. In this article, we pursue this line of thought by proposing an unconventional channel geometry, alternative to standard MHDC device configurations. The proposed geometry is based on a two-channel annular shape of the cross section, where the core channel communicates with the external annular region through slits of fixed opening, say *A*, which act as a cut-off length for particle size. Figure 2a schematically depicts the channel cross-section. The three-dimensional channel is created by alternately extruding the cross-sectional profiles in Figure 2b,c in the direction orthogonal to the picture, so that the internal channel can be held in place by the bearings slanted at 45°. Thus, the manufacturing of the device is ideally suited for 3D-nanoprinting.^28−30^

The suspension, initially injected only within the core region (grey shaded area), is pushed through the channel by a pressure-driven flow. As the suspension flows downstream the channel, particles of size smaller than *A* (represented in red) enter by diffusion the external annular channel through the communication slits, until a homogeneous distribution across the entire channel cross section (including both the core and the annular region) is achieved. Particles of size bigger than *A* (yellow) are instead confined within the core channel at all times. The ratio *h*/*H* is designed so that the core and the annular channel are characterized by significantly different average flow velocities when subject to one and the same pressure drop. Because small particles explore the channel core as well as the annular region, their average velocity is influenced by the average flow velocity in both regions. In an abstract sense, the separation mechanism at work in the proposed geometry exploits the best of SEC and MHDC features while reducing the drawbacks of each separation technique.

Likewise SEC, it enforces a cut-off length neatly separating two zones characterized by altogether different transport properties, thus creating average transport properties that are sensitively dependent on particle size in a narrow size interval about *A*. Besides, the amplification of axial dispersion is here much more contained compared to SEC columns.

As in MHDC, the equipment is characterized by a simple, ordered geometry, which allows for a precise predictability and control over particle motion. The amplification of velocity differences between particles of different size boosts the separation performance of the operation, allowing to obtain a prescribed resolution in a sensitively shorter device length than standard MHDC and, consequently, in a considerably shorter operational time. Throughout the article, we refer to the separation mechanism governing small particles in the two-channel geometry as Brownian sieving to highlight how the driving force for the separation hinges on the interaction between particle diffusion and confined geometries whose smallest length scale is comparable to particle size. Consistently, the two-channel annular geometry is henceforth referred to as BS-MHDC (Brownian sieving MHDC). We observe that the BS mechanism enforced in the proposed device acts transversally to the streamwise direction of particle motion and, as such, differs from other diffusion-based mechanisms previously discussed in the literature.^31,32^

## Experimental Section

### Device Geometry

**Figure 3:**
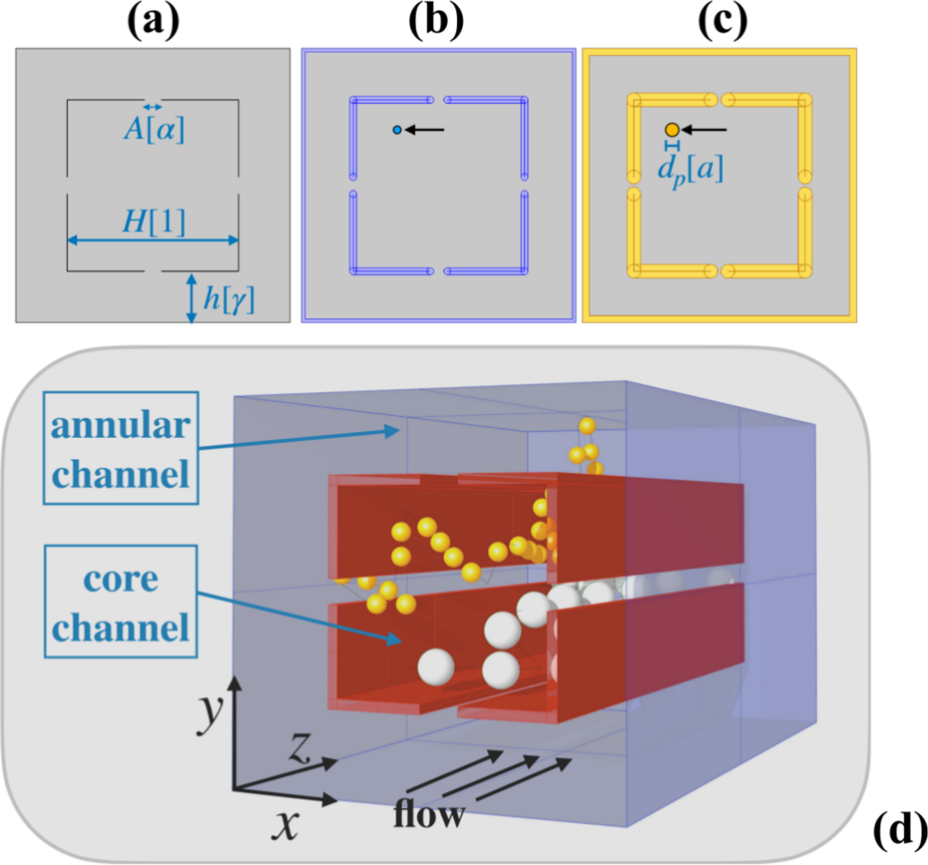
Device geometry and reference system. The gray shaded area in panels (a–c) depicts the effective transport domain, Ω_*d*_p__, accessible to the center of mass of a supposedly spherical particle of diameter *d*_p_ for a point tracer (*d*_p_ = 0) and two particles of increasing size whose diameter is below the size, *A*, of the communication slit. The lettering between square brackets denotes the dimensionless length with respect to the reference length *H*. Panel (d) shows the three-dimensional structure of the channel and the qualitative structure of particle motion for two particles of diameter above (gray) and below (yellow) the cut-off length α.

In what follows, we consider an idealized version of the scheme depicted in Figure 2, where we assume vanishing thickness of the internal baffles delimiting the core channel. Figure 3a shows the system cross section with the relevant lengths defining the geometry. The lettering between square brackets denotes the corresponding dimensionless quantity, scaled with respect to the core channel width, *H*, which is next assumed as the reference length.

The channel is extruded in the direction of its axis, as represented in Figure 3c, which also fixes the reference frame used throughout the remaining of the article. The gray shaded area in Figure 3 represents the *xy* projection, Ω_*d*_p__, of the transport domain that is accessible to the center of mass of a spherical particle of diameter *d*_p_. Specifically, panels (a–c) of the figure denote the cross-sectional domain Ω_0_ accessible to a point tracer (*d*_p_ = 0) and those associated with particles of increasing diameters, respectively. Henceforth, we denote by ∂Ω_*d*_p__ the boundary of the size-dependent effective transport domain Ω_*d*_p__. Note that in the case of a point-sized particle, the cross-sectional boundary ∂Ω_0_ reduces to the walls of the square channel and the internal baffles, which have been assumed of vanishing thickness.

By following a one-way coupling approach, as discussed in the Introduction, setting up the particle transport model consists of two separate steps, namely, (i) computing the pressure-driven single-phase flow of the suspending fluid through the channel and (ii) defining the governing equations for the dynamics of a generic particle. These points are next discussed separately.

### Eluent Flow

**Figure 4:**
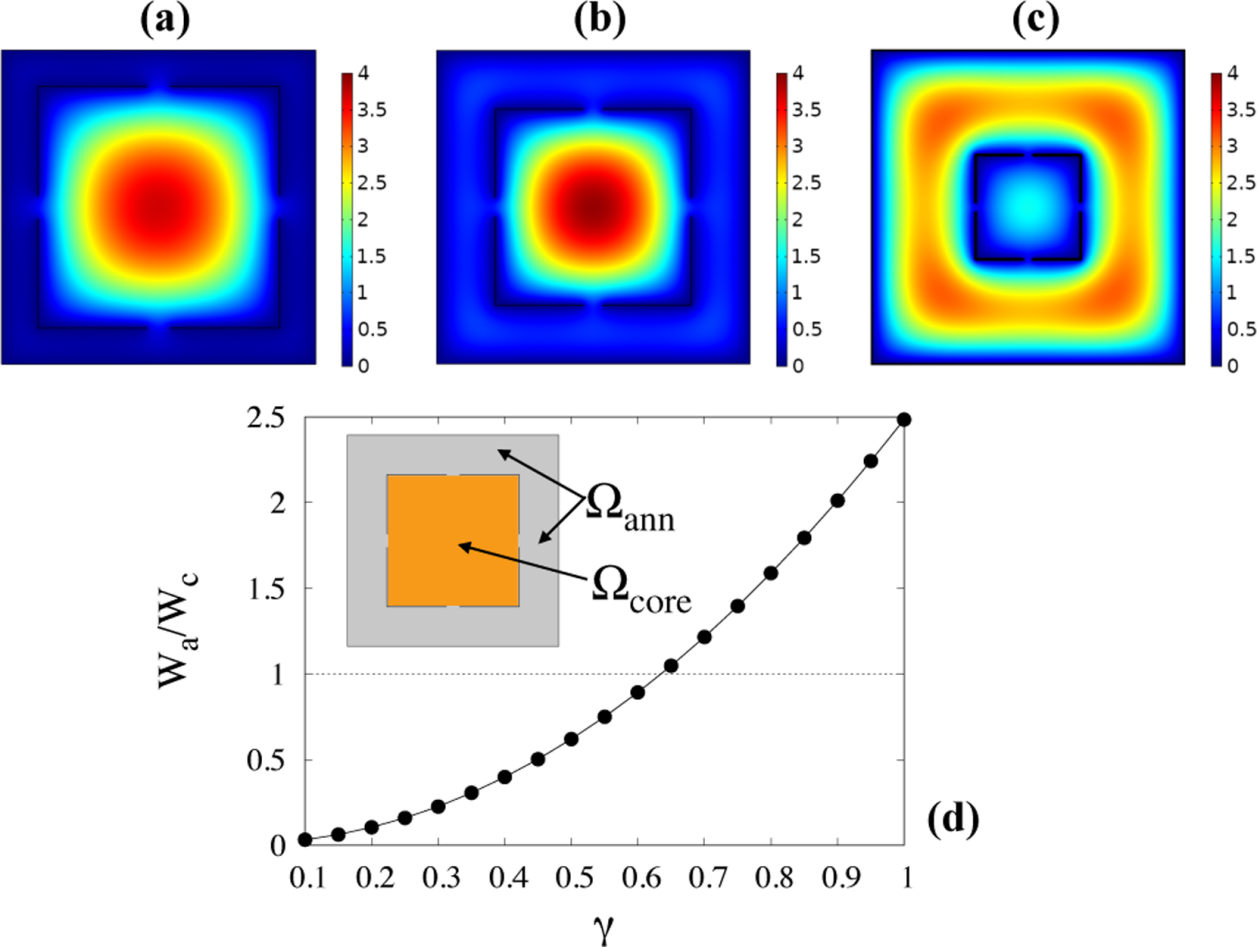
Panels (a) through (c) velocity contour of the purely axial Stokes flow in the two-channel geometry for α = 0.1 and (a) γ = 0.15, (b) γ = 0.3, (c) γ = 1. Note how the magnitude of the velocity in the annular region increases when increasing the dimensionless thickness of the annular channel γ. Panel (d) ratio of the average flow velocities *W*_a_ and *W*_b_ associated with the core, Ω_core_, and annular region, Ω_ann_, respectively.

The fluid-dynamics of the system is defined onto the three-dimensional domain , obtained extruding the transport domain Ω_0_, associated with point-sized particles, by the overall length of the channel *L*. The boundary of  consists of the inlet and outlet cross section of the device, together with the internal baffles and the channel walls. In practical implementations of MHDC, the ratio between the channel length to the characteristic size of the cross section *H* is very large, for example, order 10^5^ ÷ 10^8^. Under these conditions, the steady laminar flow through the channel can be regarded unidirectional, thus possessing only one nonvanishing (axial) component *w*, which depends solely on the cross-sectional coordinates. The axial velocity component is obtained by solving a two-dimensional Poisson problem (see Section S1.1 of the Supporting Information). In the remainder of the article, lengths are made dimensionless with respect to the edge of the core channel *H*. The dimensionless width of the communication slit and the ratio of the edge of the internal channel to the width of the annular external channel will be denoted by α = *A*/*H* and γ = *h*/*H*, respectively (see Figure 2). Figure 4a,b,c depicts the axial velocity profile for α = 1/10 and three values of γ, γ = 0.15; 0.3; 1. One notes how the magnitude of the axial velocity increases when the relative thickness of the external annular channel is increased with respect to the internal core.

A more quantitative representation of the effect of γ on the velocity profile can be gained by defining the average flow velocities *W*_c_ = (1/*S*_core_)∫_Ω_core__*w*(*x*,*y*) d*x*d*y* and *W*_a_ = (1/*S*_ann_)∫_Ω_ann__*w*(*x*,*y*) d*x*d*y*, in the core and annular regions, respectively. Here, *S*_core_ and *S*_ann_ represent the area of the regions Ω_core_ and Ω_ann_, respectively, represented in the inset of Figure 4d.

Figure 4d reports the ratio of the annulus to the core average flow velocities at increasing values of the parameter γ for a value α = 0.1 of the slit width. The lowest value for γ has been chosen equal to α which represents the largest particle size allowed to access the annular channel. By the discussion above about the Brownian sieving separation mechanism, a sizeable enhancement of separation performance of the BS-MHDC with respect to St-MHDC (i.e. single channel MHDC) is expected when the ratio *W*_a_/*W*_c_ departs significantly from unity (a condition that for the chosen value of α is obtained at γ* = 0.635). For γ smaller than this value, the average flow velocity in the annular region is smaller than that in the core channel, and particles accessing this region will travel on the average at lower velocity than those confined to the core channel. Thus, the dependence of average particle velocity on particle size is qualitatively consistent with that observed in standard MHDC; that is, the average particle velocity increases with particle size. Besides, for γ > 0.635, the average velocity in the annular region is greater than that in the core. In these conditions, an *inversion* of the separation drive is expected with respect to standard MHDC, in which smaller particles will attain a larger average velocity with respect to the bigger particles, and therefore, they will be eluted first. Next, we introduce the particle transport model useful to put these heuristic observations onto firm quantitative grounds.

### Particle Transport Approach

Let us then define the governing equation for the dynamics of a generic particle which accounts for fluid drag, wall-hindrance effects, and Brownian diffusion. Regarding the (Stokesian) drag of the fluid, we assume that the particle is in the overdamped regime,^33^ which amounts to setting the instantaneous particle velocity equal to the velocity of the unperturbed single-phase flow computed at the particle center of mass. Particle diffusion is accounted for by adding a stochastic component to the deterministic displacement associated with the Stokesian drag. This leads to considering a Langevin-type stochastic differential equation for the dynamics of a generic particle, which in the dimensionless form can be written as
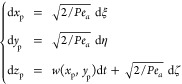
2where *x*_p_, *y*_p_, and *z*_p_ denote the dimensionless coordinates yielding the position of the center of mass of the particle, scaled with respect to the reference length *H* (see Figure 3a), *t* is the dimensionless time, scaled with respect to the convective reference time τ_c_ = *H*/*W*, *W* being the average flow velocity over the entire device cross section, and where *w*(*x*_p_, *y*_p_) is the axial velocity solution of the Poisson problem made dimensionless with respect to *W*. The dimensionless particle Péclet number , appearing at the right hand side of eq 2, represents the ratio of the characteristic diffusion timescale  ( being the diffusion coefficient of the particle), to the convective timescale τ_c_. For a spherical particle, the bare particle diffusivity  can be estimated through the Stokes–Einstein relationship as , where *k*_B_ is the Boltzmann constant, *T* the absolute temperature, μ the dynamic viscosity of the fluid, and *d*_p_ the (dimensional) particle diameter. It is worth observing that *Pe*_*a*_ implicitly depends on the particle diameter through the particle diffusion coefficient. The fluctuating components dξ, dη, and dζ in eq 2 are the increments of a Wiener process characterized by zero mean and unit variance, whereas the symbol *a* appearing as a subscript of the Péclet number represents the dimensionless particle diameter, *a* = *d*_p_/*H*. The Langevin equation is advanced in time by a finite time increment Δ*t* using the Euler–Maruyama algorithm,^34^ enforcing elastic collisions at the boundaries (see Section S1.2 of the Supporting Information for details). The approach described above has been successfully applied to quantify transport of diluted suspensions in a wealth of microfluidic devices and has been recently validated against available experimental data in complex device geometries.^35^ It should be observed, however, that a wealth of different types of wall–particle interactions may arise depending on the nature of the suspended objects, of the suspending solution and of the solid boundaries, which range from EDL-driven electrostatic effects to more complex scenarios, such as those exploited for steering self-propelled colloidal particles.^36,37^ In the next Section, eq 2 is used to investigate the dynamics of particle ensembles in the BS-MHDC device.

## Results

For all of the results next discussed, the initial positions of the center of mass of the particles are uniformly distributed over the allowed region of the internal (core) channel at the device entrance, that is, *z* = 0. Starting from the initial positions, *N*_p_ = 10^5^ particles are advanced in time, and the position of their center of mass is recorded at regular time intervals. The dimensionless width, α = *A*/*H* (see Figure 3a), of the communication slit between the core and the annular channel is set to α = 0.099, whereas γ = 0.3 is chosen as a representative value ensuring significantly different velocities between the annular and the core channel (see Figure 4d) so that the Brownian sieving mechanism is expected to induce sizeable differences between the dynamics of particles whose size falls below and above the cut-off length α.

### Dynamics of Monodispersed Particle Ensembles in BS-MHDC

**Figure 5:**
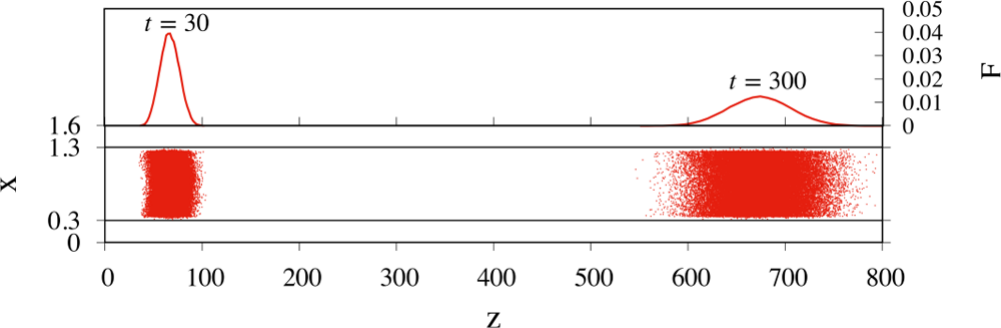
Bottom panel: projection onto the *xz* plane of the instantaneous position for an ensemble of 10^5^ particles initially distributed uniformly onto the cross section of the core channel. The positions at dimensionless times *t* = 30 and *t* = 300 are depicted. The channel geometry is specified by α = 0.099 and γ = 0.3. The particle radius is set to *a* = 0.1, just above the cut-off length α so that particles are prevented from entering the annular low-velocity channel, which, in the *xz* projection of the figure, is represented by the two regions 0 ≤ *x* ≤ 0.3 and 1 ≤ *x* ≤ 1.3. The panel at the top of the channel projection shows the marginal probability density function *F*(*z*; *t*).

Let us first consider the dynamics of particles larger than the slit width α, which are confined by hindrance effects to the core channel. Figure 5 (bottom panel) shows the projection of particle positions onto the *xz* plane. Snapshots are taken at (dimensionless) times *t* = 30 and *t* = 300 for a value *Pe*_*a*_ = 10^2^ of the particle Péclet parameter. The (dimensionless) particle diameter *a* is here set to *a* = 0.1, just above the width of the slit opening α.

**Figure 6:**
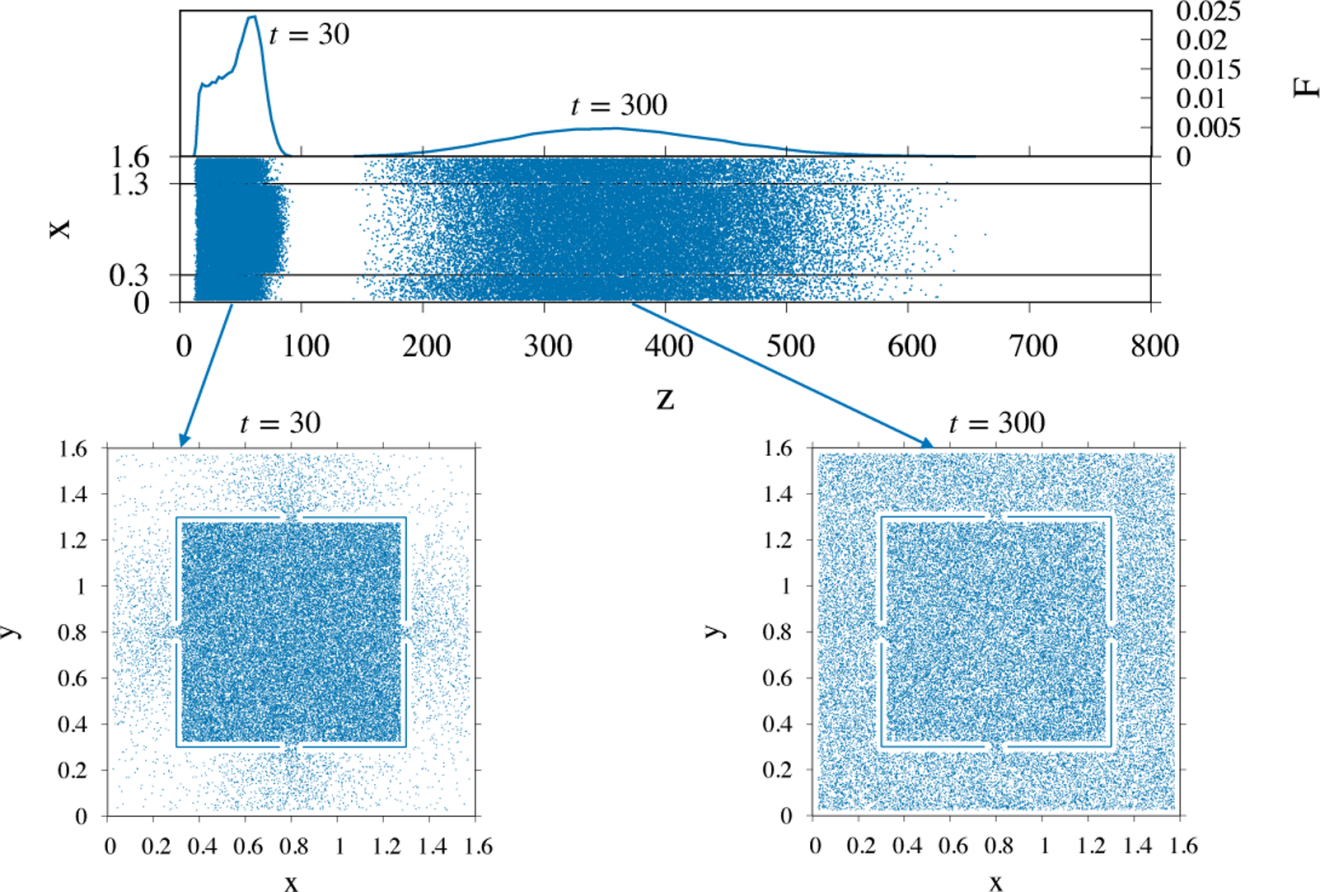
Particle dynamics for the same geometry depicted in Figure 5 for a particle diameter *a* = 0.05 at *Pe*_a_ = 50. At the early stage of the process (*t* = 30), the marginal distribution *F*(*z*; *t*) has not yet attained a symmetric Gaussian shape. The Gaussian distribution is recovered only at later times (*t* = 300), when the particles are spread uniformly across the entire cross section of the two-channel geometry. The bottom panels show the cross-sectional projection of the particle center for the ensemble at the corresponding times.

The top panel of the figure depicts the marginal probability density function (PDF) *F*(*z*; *t*) of the *z* coordinate of the particle center of mass. Therefore, *F*(*z*; *t*) d*z* yields the fraction of particles whose center falls between *z* and *z* + d*z* at time *t*, regardless of the particle position onto the channel cross section. Note how, on the average, particles travel faster than the eluent (whose average velocity is unitary in the dimensionless setting). This is because they only sample the flow in the core channel, where the average velocity of the fluid restricted to the core cross section, *W*_c_, is larger than unity (compare with Figure 4b,d). The transport regime for the suspended particles can be quantitatively characterized by considering the mean, *z*_c_(*t*), and the variance, σ(*t*), of the marginal distribution *F*(*z*; *t*), which are defined by  and , where the superscript “(*h*)” identifies the individual particle of the ensemble. Likewise point tracers, after an initial transient behavior, finite-sized particles are eventually expected to attain a macrotransport regime^22^ characterized by the scaling laws
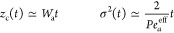
3where *W*_a_ is henceforth referred to as the (dimensionless) *average particle velocity* and 1/*Pe*_*a*_^eff^ as the (dimensionless) *effective dispersion coefficient*. For the case depicted in Figure 5, one obtains *W*_a_ = 2.25 and 1/*Pe*_*a*_^eff^ = 1.71. Thus, particles of this size travel more than twice as fast as the average flow and are characterized by a dimensionless dispersion coefficient two orders of magnitudes bigger than the dimensionless bare particle diffusivity 1/*Pe*_*a*_ = 10^–2^. Let us next shift our focus to the dynamics of particles of size below the opening width α. Figure 6 shows the simulation results for the same geometry and operating conditions as those of Figure 5 for particles of dimensionless diameter *a* = 0.05, which can enter the annular region. Note that with all other parameters left unchanged, the particle Péclet number, , (where  is the particle diffusivity) scales linearly with the particle diameter. Therefore, if the numerical experiment next discussed is to represent particles of dimensionless size *a* = 0.05 entrained in the same flow system as that considered in Figure 5, then, *Pe*_*a*_ for this particle size must be set to *Pe*_*a*_ = 50 in that the ratio of the two Péclet numbers must be equal to the ratio of particle diameters, that is, *Pe*_0.05_ = *Pe*_0.1_/2.

From the data reported, one observes how, for this particle size, the short time behavior of particle dynamics is characterized by a marginal distribution *F*(*z*; *t*) whose shape departs from the symmetric Gaussian template. This is because the particles are initially placed in the internal core channel, and a finite amount of time is necessary for reaching the uniform particle concentration across the allowed cross-sectional area ∂Ω_a_, as can be gathered from the bottom panels of the figure. In this time interval, the mean and variance of *F*(*z*; *t*) do not strictly follow eq 3 (see Section S2.1 of the Supporting Information). The asymptotic scaling of the variance represented in Figure S3 of the Supporting Information yields an effective dispersion coefficient almost 3 orders of magnitude larger than the bare particle diffusivity, an occurrence that hinders separation resolution. Besides, as discussed below, the positive effect of the Brownian sieving mechanism on the effective particle velocity largely overcomes the negative impact of the amplified dispersion.

### Comparison of the Separation Performance between St-HCD and BS-MHDC

**Figure 7:**
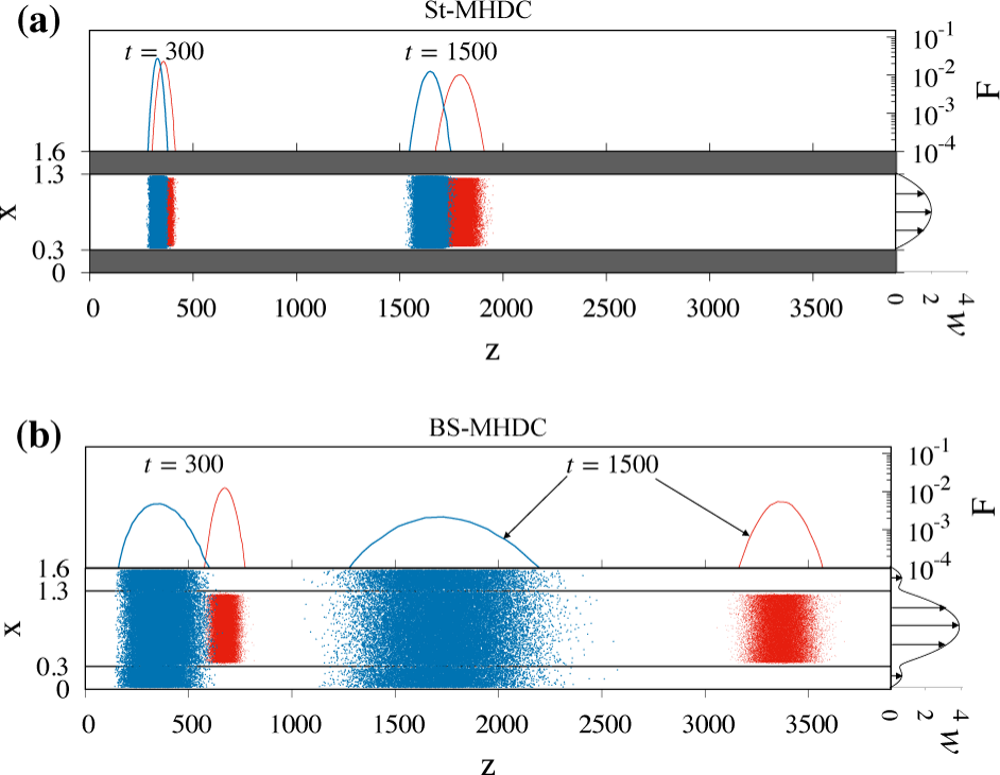
Comparison between (a) St-MHDC and (b) BS-MHDC at *Pe*_0.1_ = 10^2^ for particles of dimensionless diameter *a* = 0.1 (red) and *a* = 0.05 (blue). Particles of both sizes are initially uniformly distributed onto the cross section of the core channel at *z* = 0. In St-MHDC (a), both particles are confined to the core channel (α = 0) and cannot enter the annular region (grey shaded area). In the two-channel configuration (α = 0.099), the smaller particles can access the annular region. The (dimensionless) average flow velocity is equal in the two cases, *W* = 1. The scale of the marginal distribution *F*(*z*; *t*) has been chosen logarithmic for visualization purposes. The curve with vectors at the channel exit depicts the velocity profile along the vertical symmetry line of the cross section, *w*(*x*,*y* = 0.8).

Next, we compare the separation performance of the BS-MHDC geometry enforcing the Brownian sieving effect with that of standard MHDC, henceforth referred to as St-MHDC, which we define as a channel with squared cross section with the edge length equal to *H* with impermeable boundary (i.e. α = 0). Thus, in the dimensionless formulation, the cross section of the St-MHDC is the unit square. Note that because of the presence of internal baffles in the BS-MHDC geometry, conditions corresponding to the same average flow velocity in the two systems correspond to different values of the overall pressure drop. In what follows, we define the operating conditions based on the *Pe* value of the particle of size *a* = 0.1, henceforth denoted with *Pe*_0.1_. Figure 7 shows the comparison between the separation performance of St-MHDC (Figure 7a) and BS-MHDC (Figure 7b) at *Pe*_0.1_ = 100.

By the choice of the reference velocity in the dimensionless formulation of the two thought experiments, the (blue) particles are characterized by close values of the average particle velocity, as can be gathered by observing that the peaks of the marginal distributions are located at essentially the same *z* coordinate at corresponding times. One notes how the dispersion bandwidth characterizing the smaller particles in the two-channel geometry is sizeably amplified by the Brownian sieving effect with respect to the standard MHDC geometry. However, the comparison makes it evident that a complete resolution of the two-particle mixture in the BS-MHDC channel is accomplished already at time *t* = 300, whereas the marginal distributions of the small and large particles are still partially overlapped in the St-MHDC geometry at time *t* = 1500. This example clearly indicates that the effect of the Brownian sieving mechanism in amplifying the difference of the average particle velocity for the two sizes largely overcomes the effect of the band broadening caused by the augmented value of the dispersion coefficient 1/*Pe*_0.05_^eff^ observed for the smaller particles. Because the variance of the marginal distribution *F*(*z*; *t*) for each particle size depends on the particle Péclet number, a natural question arises as to how the efficiency enhancement is influenced by the operating conditions, here quantified by the *Pe*_0.1_ value. Figure S4 of the Supporting Information shows that the separation enhancement induced by the Brownian sieving is even further increased with respect to the case shown in Figure 7 when the reference Péclet value is decreased by a factor 10 (e.g., with all other conditions left unchanged, the eluent velocity is lowered by a tenfold factor).

**Figure 8:**
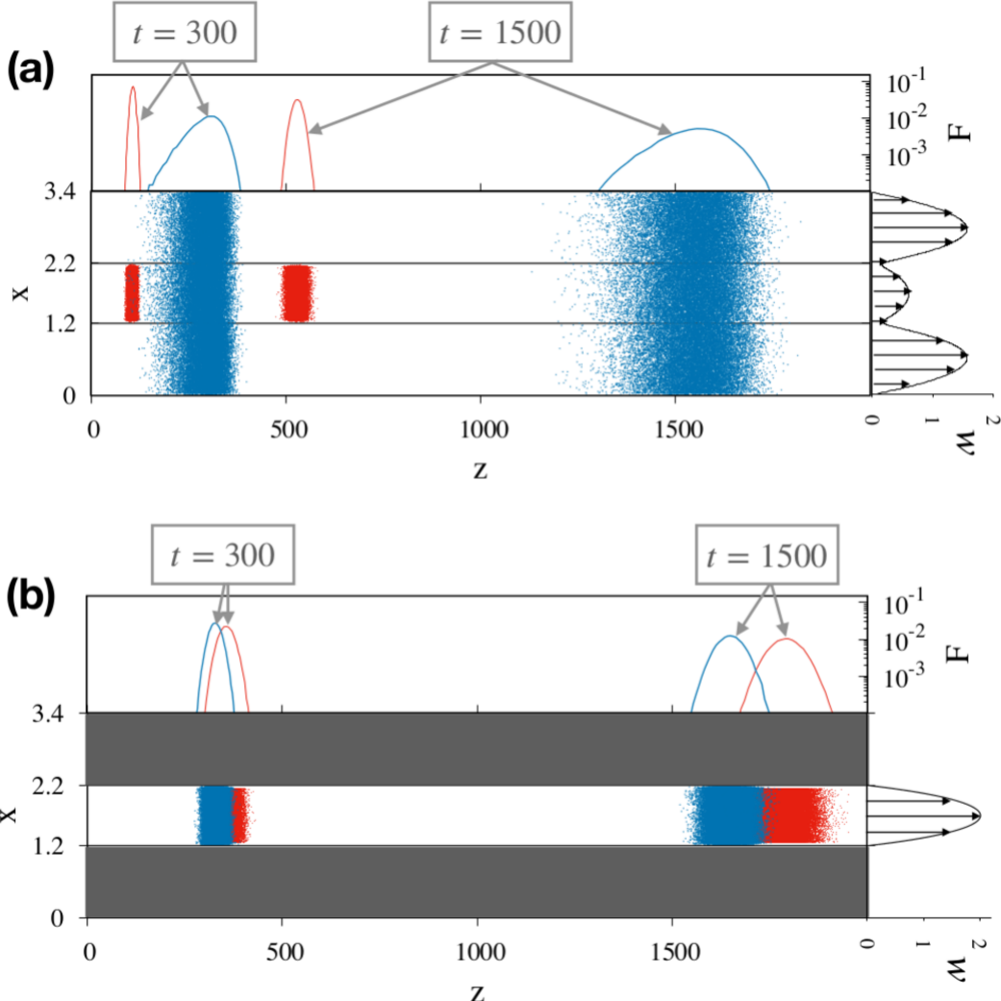
Comparison between (a) BS-MHDC and (b) St-MHDC for γ = 1.2, at *Pe*_0.1_ = 10^2^ for particles of dimensionless diameter *a* = 0.1 (red) and *a* = 0.05 (blue). All other parameters are the same as those of Figure 7.

An interesting phenomenology arises when the value of the parameter γ is chosen greater that the critical value, γ = 0.635, associated with equal average flow velocities *W*_a_ and *W*_c_, that is, when *W*_a_ > *W*_c_. Figure 8 depicts the evolution for the two-size particle mixture at γ = 1.2.

Here, the classical dependence of the average particle velocity on particle size characterizing St-MHDC is inverted. The small particle ensemble (blue) travels at a larger velocity downstream the channel and will be eluted before the bigger particles. Owing to a larger characteristic diffusional length associated with Ω_ann_ at γ = 1.2, the probability density function *F*(*z*; *t*) of the small particles maintains asymmetric features even at dimensionless time *t* = 1500. However, a sizeable enhancement of separation efficiency is also evident in the BS-MHDC device for this choice of the geometric parameter γ, in which a complete resolution of the particle mixture is already achieved at *t* = 300, whereas the two-size mixture is still unresolved in the St-MHDC at *t* = 1500.

## Discussion

In order to gain a quantitative overview of the BS-MHDC performance, let us introduce the resolution, *R*(*t*), associated with a mixture of particles “1” and “2” of size *a*_1_ and *a*_2_
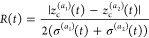
4

The condition requiring *R*(t*) = 1 defines the minimum time t* for the resolution of the mixture, and consequently, the minimum channel length ensuring that no significant overlap of the particle distribution exists at the exit of the channel. At large times, that is, when macrotransport conditions have been reached for each particle ensemble, it is expected that . This is because, from eq 3, one derives that the numerator at the right hand side of eq 4 grows linearly with time (with rate |*W*_*a*_1__ – *W*_*a*_2__|), whereas the variances at the denominator grow as . The behavior of *R*(*t*) versus *t* for different geometries and different values of *Pe*_0.1_ is depicted in Figure S5 of the Supporting Information.

**Figure 9:**
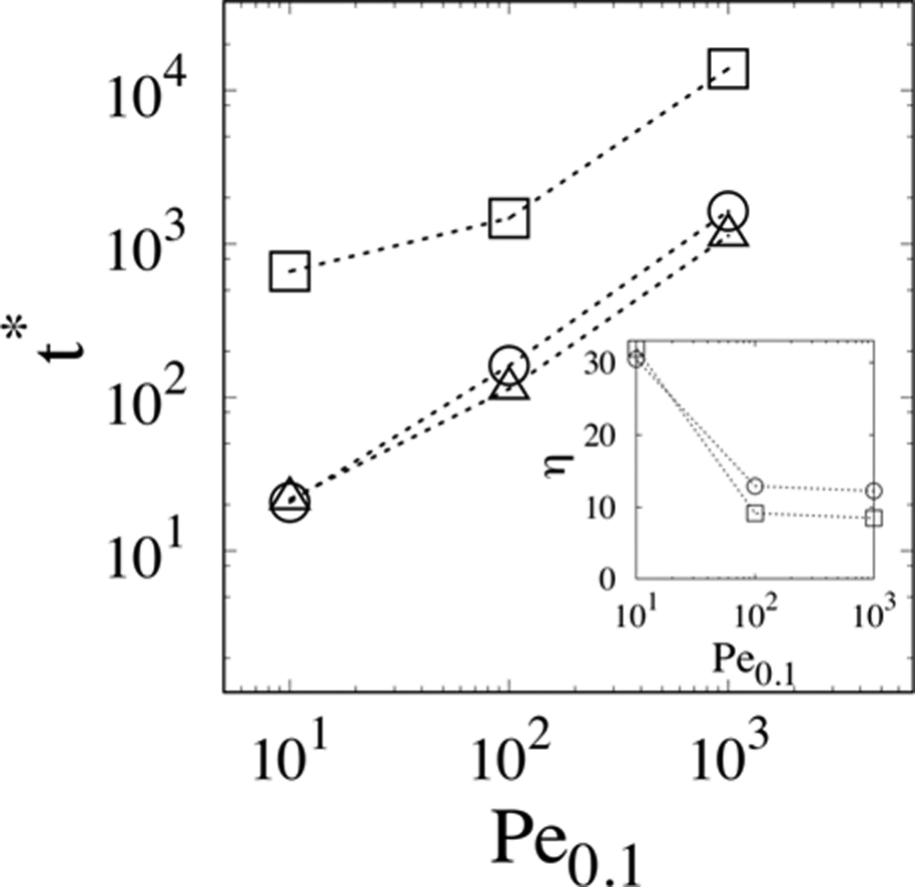
Main panel: resolution time, t*, vs *Pe*_0.1_. Squares: St-MHDC; circles: BS-MHDC (γ = 0.3); triangles: BS-MHDC (γ = 1.2). The inset depicts the enhancement factor between St-MHDC and BS-MHDC for γ = 0.3 (squares) and for γ = 1.2 circles.

A quantitative measure of the improved separation performance can be obtained by considering the (dimensionless) resolution time, t*, and by defining the enhancement factor, η, between St-MHDC and BS-MHDC geometries as the ratio of resolution times
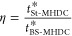
5which is also equal to the ratio between the channel lengths ensuring complete separation of the two-particle mixture at the device outlet. The dependence of t* on *Pe*_0.1_ in the three device geometries is shown in the main panel of Figure 9.

As expected, the value of t* increases with *Pe*_0.1_ for all device geometries and that associated with the St-MHDC (square symbols) is sensitively higher than those of the BS-MHDC geometries (circles and triangles). The best performing geometry is that characterized by γ = 1.2 where the inversion of average particle velocity occurs, which provides the lowest value of t* at moderate and high *Pe*_0.1_. The inset of Figure 9 depicts the enhancement factor η between St-MHDC and BS-MHDC for γ = 0.3 (squares) and for γ = 1.2 (circles). At low values of the Péclet parameter, η is of order 30 in both BS-MHDC geometries. This means that the enforcement of the Brownian sieving mechanism allows to achieve a complete separation of the two-particle mixture within a device thirty times shorter than St-MHDC at the same average velocity of the eluent. Also, it is interesting to notice how the enhancement factor is well above unity in the whole range of Péclet values considered, which covers the vast majority of conditions encountered in practical implementations of MHDC-based separations. This implies that the enhancement induced by the Brownian sieving is robust to changes of the operating conditions, such as, for instance, to increasing/decreasing the eluent velocity. The working principle above discussed for two particle sizes can readily be adapted to the fractionation of an arbitrary size-distribution of particles by making the slit opening α dependent on *z* (see Section S4 of the Supporting Information). Therefore, it could become competitive with other size-based separation techniques, such as, deterministic lateral displacement.^38^

## Conclusions

One major limitation of MHDC methods for the size-based separation of particle suspensions and colloids is represented by their low efficiency, ultimately stemming from the fact that the maximum relative difference between the average velocity of particles of different size is of the order of few percents. We propose an improved MHDC device constituted by a two-channel annular geometry, where the core and the external annular channel communicate through slits of assigned width. By appropriately tuning the ratio between the thickness of the annular channel and the width of the core channel, two separated zones of the cross section can be created, which are characterized by altogether different values of the average flow velocity when subjected to the same pressure drop. Particles smaller than the slit width can enter the annular channel by transversal diffusion and sample the average velocity of both channels, whereas particles bigger than the cut-off length are confined to the core channel, so that their velocity is influenced only by the flow in this region. We call this effect Brownian sieving. We investigate the separation performance of a two-particle mixture through a Lagrangian stochastic model for particle motion, which accounts for fluid drag, Brownian diffusion, and wall hindrance effects. The analysis of two selected geometries enforcing the BS-MHDC mechanism suggests that the enhancement factor induced by the Brownian sieving effect with respect to the standard MHDC geometry can reach values well above ten, depending on the operating conditions here characterized by the particle Péclet number. We expect that the enhancement factor can be further improved if an optimization of the device geometry and of the working *Pe* value to maximize the separation resolution is carried out. Besides, this type of analysis cannot be performed through Lagrangian methods in view of the large CPU time involved in the simulation of the particle ensemble dynamics and can be conveniently approached through Brenner’s macrotransport paradigm. This study will be the object of future work.

## Supporting Information Available

Eluent flow; numerical approach for particle transport; dynamics of monodispersed particle ensembles in BS-MHDC; comparison between St-MHDC and BS-MHDC; discussion; and multistage geometry (PDF)
