# Supplementary material for: Brownian Sieving Effect for Boosting the Performance of Microcapillary Hydrodynamic Chromatography. Proof of Concept
Source: Anal Chem. 2021 Apr 23;93(17):6808–16. doi: 10.1021/acs.analchem.1c00780 (PMC8253478; doi:10.1021/acs.analchem.1c00780)
Supplement: Supplementary file 1 — ac1c00780_si_001.pdf [file ac1c00780_si_001.pdf]

SUPPORTING INFORMATION
Brownian sieving effect for boosting the performance of microcapillary hydrodynamic chromatography. Proof of concept.

Valentina Biagioni, Alpha L. Sow, Alessandra Adrover, and Stefano Cerbelli*

Dipartimento di Ingegneria Chimica Materiali Ambiente, Sapienza Università di Roma - Via Eudossiana 18 - 00184 Roma (Italy)

E-mail: stefano.cerbelli@uniroma1.it

Contents

1 Numerical solution of the transport model **S2**

1.1 Eluent flow S2

1.2 Numerical approach for particle transport S3

2 Results **S5**

2.1 Dynamics of monodispersed particle ensembles in BS-MHDC S5

2.2 Comparison between St-MHDC and BS-MHDC S7

3 Discussion **S9**

4 Multistage geometry **S11**

References **S11**

1 Numerical solution of the transport model

1.1 Eluent flow

The axial component $w(x, y)$ of the Stokes flow $\mathbf{v} = (u(x, y, z), v(x, y, z), w(x, y, z)) = (0, 0, w(x, y))$ is the solution of the two-dimensional Poisson equation

$$\nabla_\perp w(x, y) = \frac{1}{\mu}\frac{\partial P}{\partial z} \qquad (1)$$

where $\nabla_\perp = \partial^2/\partial x^2 + \partial^2/\partial y^2$ is the cross-sectional Laplacian operator, μ is the dynamic viscosity of the suspending solution, and $\partial P/\partial z = -\big(P|_{z=0} - P|_{z=L}\big)/L$ is the pressure gradient in the axial direction. Equation (1) is equipped with the no-slip/impermeability boundary conditions, $\mathbf{v} = 0$, on all of the solid surfaces delimiting the double channel, i.e. for $(x, y) \in \Omega_0$ and $0 \le z \le L$. Unlike the case of open channels of cylindrical or rectangular shape, a closed-form solution of the Poisson boundary value problem is not available because of the presence of the internal baffles where no slip conditions apply. Besides, due to the two-dimensional nature of the problem and to the linear character of Eq. (1), the numerical solution of the flow poses no severe challenge. For all of the results showed next, the solution of the two-dimensional Poisson problem in Eq. (1) has been obtained by a commercial finite-element software (Comsol Multiphysics 5.5) by using an unstructured mesh corresponding to order 10^5 degrees of freedom.

1.2 Numerical approach for particle transport

The Euler-Maruyama algorithm for the integration of the Langevin equation (Eq. (4) of the main text) reads

$$\begin{cases} x_p(t+\Delta t) &= x_p(t) + \sqrt{2\Delta t/\mathrm{Pe}}\,\xi \\ y_p(t+\Delta t) &= y_p(t) + \sqrt{2\Delta t/\mathrm{Pe}}\,\eta \\ z_p(t+\Delta t) &= z_p(t) + w\big(x_p(t), y_p(t)\big)\,\Delta t + \sqrt{2\Delta t/\mathrm{Pe}}\,\zeta \end{cases} \tag{S1}$$

where ξ η, and ζ are normally distributed random variables.

Because of the presence of the diffusive terms, Eq. (S1) must be supplemented with boundary conditions at the internal baffles and the channel walls, which define the interaction between the solid boundaries and the advecting-diffusing particle whenever the particle center crosses the boundaries of the effective transport domain Ω_{d_p}. Here, elastic collisions are enforced, so that the z-component of the displacement is unaffected by the collision, whereas the cross-sectional component of the displacement (convective plus diffusive) that falls beyond the effective boundary $\partial\Omega_{d_p}$ is reflected along the local normal direction to the surface, as depicted in Fig. S1. Note that multiple reflections are possible, especially near the corners of $\partial\Omega_{d_p}$. In the limit where $\Delta t \to 0$ in Eq. (S1), it can be shown that the elastic collision model becomes the Langrangian equivalent of the Neuman Boundary condition of vanishing normal diffusive flux at the boundary enforced on the particle number density $\phi(x, y, z, t)$ stemming from the Eulerian description of particle transport. A thorough comparison between the two approaches has been used to validate Brenner's macrotransport approach to describe transport of finite-sized particles in periodic patterns of impermeable obstacles in deterministic lateral displacement devices.[1]

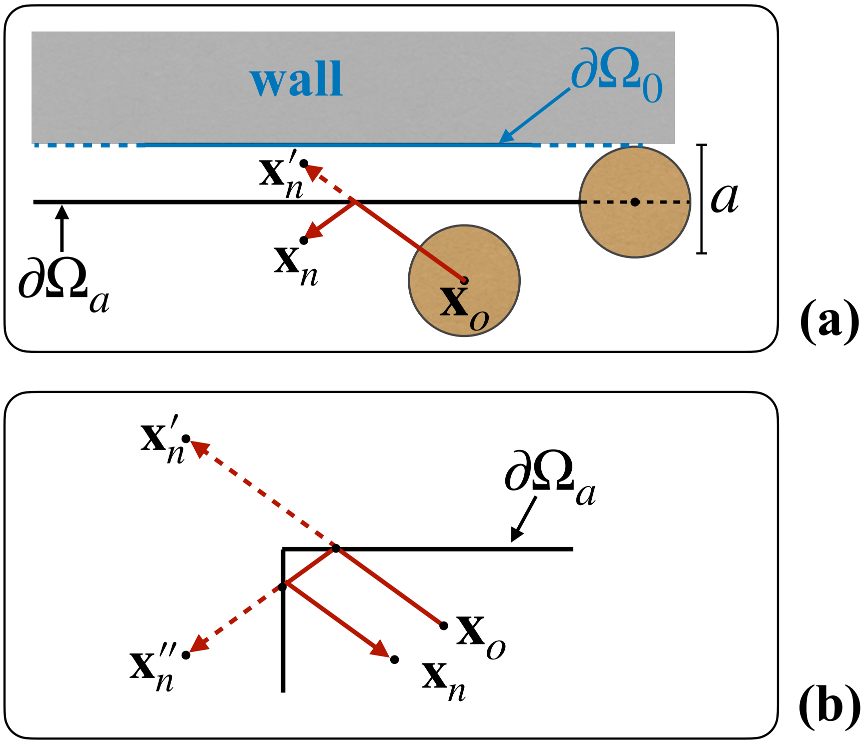

Figure S1: Reflection conditions for a particle whose center of mass crosses the boundary $\partial\Omega_{d_p}$ of the effective transport domain. Here $\mathbf{x}_o = (x_o, y_o)$ and $\mathbf{x}_n = (x_n, y_n)$ represent the position of the center of mass of the particle at time t and $t + \Delta t$, respectively. $\partial\Omega_o$ (depicted in blue) represents the physical boundary, i.e. the boundary as seen by a particle of vanishing size. Panel (a): simple reflection; Panel (b): double reflection.

2 Results

2.1 Dynamics of monodispersed particle ensembles in BS-MHDC

The asymptotic behavior of the center of mass and the variance of an ensemble of particles of dimensionless diameter α obeys the linear scalings

$$z_c(t) = K_a^{(W)} + W_a\, t \qquad \sigma^2(t) = K_a^{(D)} + \frac{2}{\mathrm{Pe}_a^{\mathrm{eff}}}\, t \tag{S2}$$

The constants $K_a^{(W)}$ and $K_a^{(D)}$ appearing in Eq. (S2) depend on the transient behaviour of the particle ensemble, before the macrotransport regime has settled in. Figure S2 shows the position $z_c(t)$ of the center of mass of the swarm and the squared variance $\sigma^2(t)$ vs. time of the particle ensemble depicted in Fig. 5 of the main text. It can be observed how, for the

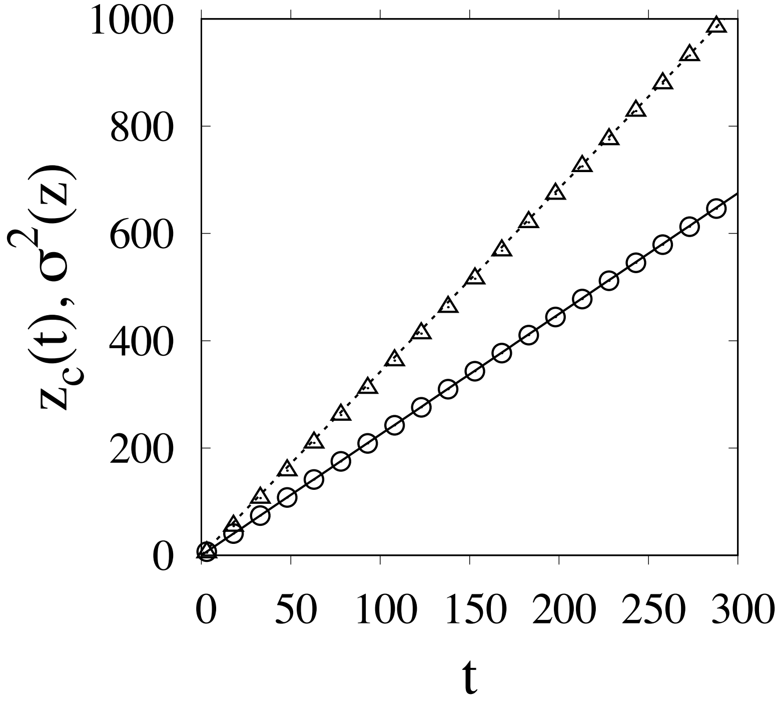

Figure S2: Average and variance of the marginal distribution $F(z;t)$ vs time for the case depicted in Fig. 5 of the main text. Circles: $z_c(t)$; Triangles: $\sigma^2(t)$. The straigth lines represent the linear fit of the data.

case considered, the linear trend characterizing the macrotransport regime manifests itself at

the very beginning of the process, i.e., $K_a^{(W)} = 0$ and $K_a^{(D)} = 0$. The continuous and broken lines represent the best linear fit to the data, which yields $W_{d_p} = 2.25$ and $1/\mathrm{Pe}_a^{\mathrm{eff}} = 1.71$ for the dimensionless average particle velocity and effective dispersion coefficient, respectively. Thus, the particles confined to the core channel travel more than twice as fast as the solvent molecules (whose dimensionless average velocity is unity). The data also provide a quantitative example of the dispersion enhancing effect resulting from the interaction of the non-uniform velocity profile and Brownian diffusion onto the channel cross-section: here, the dimensionless effective dispersion coefficient $1/\mathrm{Pe}_a^{\mathrm{eff}}$ is two orders of magnitude larger than the dimensionless bare diffusion coefficient $1/\mathrm{Pe}_a = 10^{-2}$.

As discussed in the main text, particles of radius $a = 0.05$, which can access the annular channel, display a different transient behavior of the marginal distribution $F(z;t)$. Here, a sizeable transient can be identified before macrotransport regime is attained. Figure S3 shows the behavior of $\sigma^2(t)$ at the early stage of the transport process. The square variance

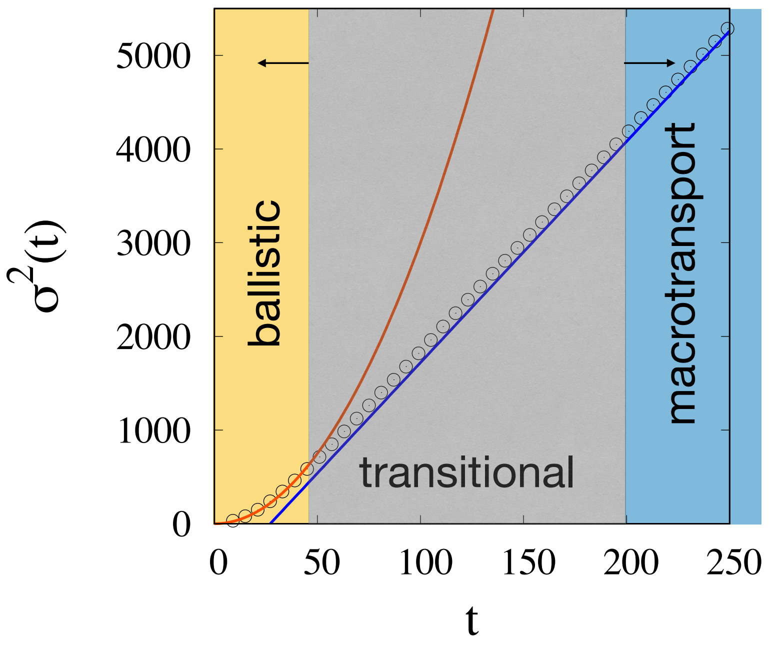

Figure S3: Short time behavior of the variance for the particle ensemble depicted in Fig. 6 of the main text. The red and blue lines line show the scalings $\sigma^2(t) = k\,t^2$, and $\sigma^2(t) = K_a^{(D)} + 2t/\mathrm{Pe}_a^{\mathrm{eff}}$, respectively.

follows an initial ballistic regime, where $\sigma^2(t) \simeq t^2$, followed by a transitional regime up to $t \simeq 200$, until macrotransport conditions are reached where the behavior of $\sigma^2(t)$ is consistent with Eq. (S2). The best fit to the data yields a value $1/\mathrm{Pe}_a^{\mathrm{eff}} = 11.8$ for the dimensionless effective dispersion, to be compared with the value $1/\mathrm{Pe}_a = 2 \times 10^{-2}$ of the dimensionless bare particle diffusivity. Thus, for this particle size at the operating conditions chosen, the dispersion enhancement factor over the bare dimensionless particle diffusivity $1/\mathrm{Pe}_a$ is almost three orders of magnitude.

2.2 Comparison between St-MHDC and BS-MHDC

Figure S4 shows the results of the comparison of separation performance at $\mathrm{Pe}_{0.1} = 10$, in the system depicted in Fig. 7 of the main text (this can be though of, e.g., as obtained by running the same experiment depicted in Fig. 7 of the main text when the eluent velocity is decreased by a factor ten, with all of the other parameters left unchanged). The comparison between Fig. 7-(a) and Fig. S4-(a) shows how the separation efficiency of the St-MHDC is weakly influenced by the mutated operating conditions, whereas that of the BS-MHDC system is sizeably enhanced (compare Fig. 7-(b) and Fig. S4-(b).).

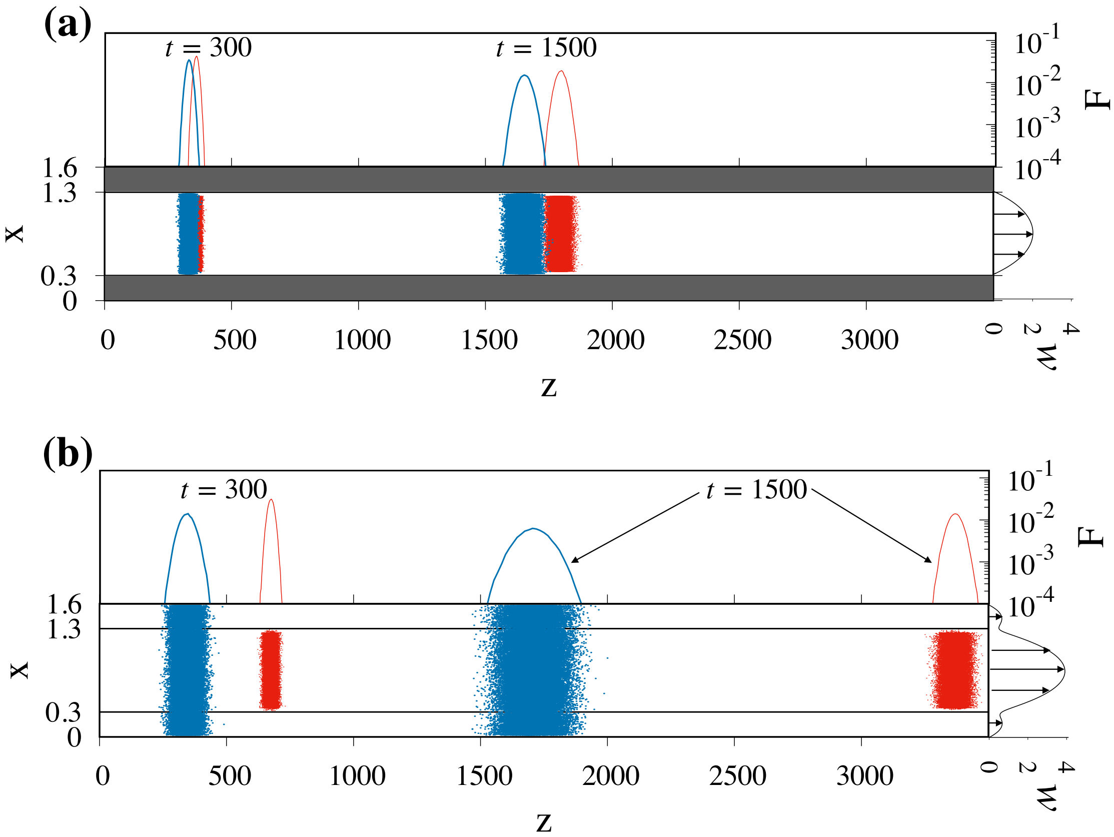

Figure S4: Comparison between -(a) St-MHDC, and -(b) BS-MHDC at $\mathrm{Pe}_{0.1} = 10^1$ for particles of dimensionless diameter $a = 0.1$ (red) and $a = 0.05$ (blue). All other parameters are the same as those of Fig. 7 of the main text.

3 Discussion

Figure S5 show the behavior of $R(t)$ vs t for the standard St-MHDC and for the two BS-MHDC geometries, $\gamma = 0.3$ and $\gamma = 1.2$ considered above over two decades of the reference Péclet value, $\mathrm{Pe}_{0.1} = 10;\ 10^2;\ 10^3$. For all of the cases considered the $R(t)$ curves associated with the BS-MHDC geometries (panel (b) and (c) of the figure) perform largely better than the standard MHDC configuration (panel (a)) in that a higher resolution is reached in the devices enforcing the Brownian sieving at corresponding times and $\mathrm{Pe}_{0.1}$ values. The comparison between $R(t)$ and the scaling $\sqrt{t}$, shown by the dash-dotted blue line, also provides a quantitative estimate of the time to reach the macrotransport regime. In the case of BS-MHDC geometry with large annular cross-section ($\gamma = 1.2$, Fig. S5-(c)) macrocransport regime is not yet reached at dimensionless time $t = 1500$ for $\mathrm{Pe}_{0.1} \geq 10^2$. The shape of the curve $R(t)$ in the transient regime is also noteworthy, as both accelerating and decelerating resolution behaviors can be observed (i.e. the first part of the curves can be either convex or concave). In the case of the St-MHDC, because the effective velocity is reached at the beginning the transport process (particles are and remain uniformly distributed over the cross-section), the shape of the curve is dominated by the behavior of the variance, which converges to macrotransport behavior at relatively short times (see Fig. S2). In the case of the BS-MHDC geometries, the analysis is more complex because the average particle velocity depends on time due to the non-uniform particle distribution onto the channel cross-section. Here, both the numerator and the denominator of the resolution factor depend non-trivially on time.

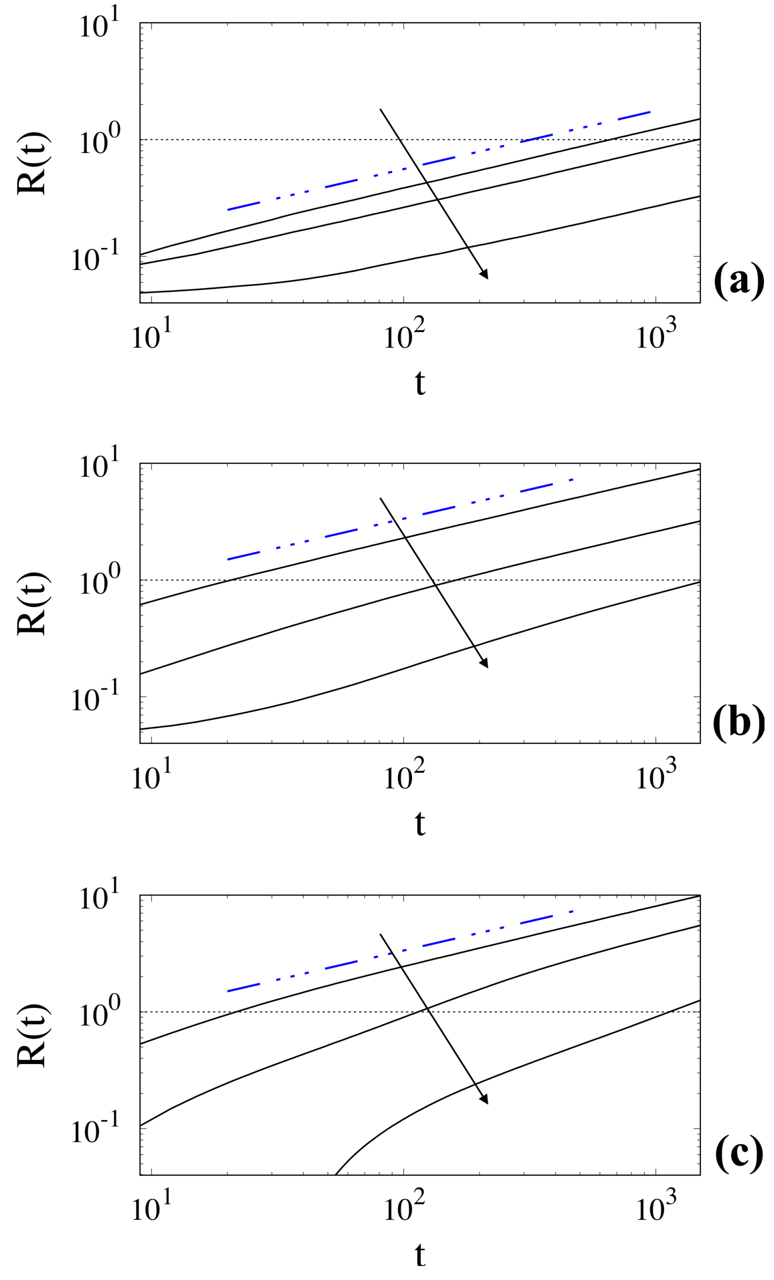

Figure S5: Resolution factor $R(t)$ vs time at $\mathrm{Pe}_{0.1} = 10$; 10^2; 10^3 ($\mathrm{Pe}_{0.1}$ increases in the direction of the arrow). The dash-dotted (blue) line depicts the scaling const. $\sqrt{t}$. The dotted line depicts the value $R = 1$, for which the mixture can be considered completely separated. (a): St-MHDC; (b): BS-MHDC ($\gamma = 0.3$); (c) BS-MHDC ($\gamma = 1.2$).

4 Multistage geometry

The separation mechanism proposed in this article can readily be generalized for the fractionation of an arbitrary size-distribution of particles. In this case, the efficient enforcement of the Brownian sieving principle can be accomplished through a device geometry where the width α of the communication slit between the core and the annular channel is made z-dependent. Specifically, one can define a set of increasing values $\alpha_1 < \alpha_2 < \cdots \alpha_n$ and assign a piecewise-constant dependence of α on prescribed intervals $z_0 = 0, z_1, z_2, \cdots z_n = L$ of the channel axis,

$$\alpha(z) = \begin{cases} \alpha_1 & \text{for} \quad 0 \leq z < z_1 \\ \alpha_2 & \text{for} \quad z_1 \leq z < z_2 \\ & \ldots \\ \alpha_n & \text{for} \quad z_{n-1} \leq z < L \end{cases} \tag{2}$$

and accomplish the fractionation of particle of increasing diameters.

References

(1) Cerbelli, S.; Giona, M.; Garofalo, F. *Microfluid. Nanofluid.* **2013**, *15*, 431–449.
